# Compliance and Performance of Hand Hygiene in Dutch General Practice Offices Using Electronic Dispensers

**DOI:** 10.1177/21501319251334218

**Published:** 2025-04-21

**Authors:** Nataliya Hilt, Matthijs S. Berends, Mariëtte Lokate, Bert Tent, Andreas Voss

**Affiliations:** 1 Department of Medical Microbiology and Infection Prevention, University of Groningen, University Medical Centre Groningen, The Netherlands; 2 Department of Medical Epidemiology, Certe Foundation, Groningen, The Netherlands

**Keywords:** primary care, electronic dispensers, compliance, hand hygiene, monitoring

## Abstract

**Introduction:**

One of the most effective measures for the reduction and prevention of healthcare-associated infections (HAI) is hand hygiene (HH). Covert direct observation of HH is difficult to realize in general practice office (GPO). The World Health Organization recognizes electronic monitoring as a form of measuring product use and estimating compliance. This is the first study to monitor HH performance electronically in Dutch GPOs.

**Objectives:**

The main aim of this study was to evaluate HH compliance in general practice offices.

**Methods:**

An observational study was conducted at 4 Dutch GPOs between 2019 and 2021. We measured HH compliance using data on HH events (HHE) from alcohol-based hand rub (ABHR) dispensers with a built-in electronic counter. Daily HH opportunities were calculated according to the ‘Five Moments for Hand Hygiene’ based on the continuously documented activities using general practitioners (GPs) patient electronic dossier systems.

**Results:**

In total, hand hygiene was performed during 1786 of the estimated 4322 opportunities (41%). HH compliance for the general practitioners, practice assistants, and nurse practitioners was 38%, 51%, and 43%, respectively. The overall HH compliance within the same GPOs was 42% pre-pandemic and rose to 56% during the pandemic. The overall mean volume of ABHR was 2.44 ml, varying per HHE between 1.91 to 2.55 ml. The mean volume of ABHR measured before and during the pandemic rose from 2.55 ml to 2.81 ml. The overall self-reported compliance was 86% and was highest among nurse practitioners.

**Conclusions:**

Hand hygiene compliance among HCWs in Dutch GPOs was found to be 41%, with general practitioners having the lowest compliance and practice assistants the highest compliance. While the mean volume of ABHR used per HHE seems appropriate, directed observations would be needed to ensure that an adequate hand-rub technique was used to cover the whole hand. Multi-modal interventions are needed to improve HH-compliance and stimulate the switch to ABHR with in the Dutch general practice office.

## Background

Health care-associated infections (HAI) pose a significant threat to patients’ safety in all domains, including primary healthcare, possibly leading to additional morbidity. Moreover, from economic and societal perspectives, infections increase costs as a result of prolonged hospital stays and additional time out of work.^1^ One of the most effective strategies for the reduction and prevention of HAIs is hand hygiene (HH).^2-4^ HH has received considerable attention during the COVID-19 pandemic as one of the fundamental interventions to prevent infections. HH compliance studies have shown that compliance rarely reaches satisfactory levels, thereby leaving patients at greater risk for infections.^5-7^ According to the World Health Organization (WHO), direct observation of HH by a trained observer is considered the gold-standard monitoring method but can be time-consuming, costly, and subject to bias.^8^ Direct observational methods also vary widely among observers and among institutions,^8,9^ making it difficult to compare the outcomes of such measurements.^10^ Also, human observers have limited capacity in terms of the number of rooms/workers they can physically observe at 1 time, particularly within large institutions.^11^

Covert direct observation of HH is difficult to realize in general practice office (GPO). To minimize the potential Hawthorne effect,^12^ healthcare workers (HCWs) must remain unaware that HH is the specific focus of observation. An alternative to the estimation of compliance with HH in GPOs can be the use of automated HH monitoring technologies. The WHO recognizes electronic monitoring as a form of measuring product use and estimating compliance, although these methods have not been fully validated, the use of electronic dispensers allows real-time documentation of hand hygiene events (HHE) without increasing the workload,^8,13^ Automatic HH monitoring systems allow the identification of correlations between compliance and its influencing factors; in 2017, an inverse association of HH compliance and workload has been demonstrated.^13^ Each type of electronic dispenser has advantages and disadvantages in terms of efficacy, accuracy, cost, and acceptance. New touchless dispensers not only reduce the risk of contamination but could also increase the frequency of use. A comprehensive study at a medical intensive care unit in Germany confirmed the positive effect of automatic dispensers on compliance, with an observed increase in hand disinfection of more than 50%.^14^ Despite this, HCWs may have concerns about privacy, which can result in a low acceptance of electronic dispensers.^15^

Building on this background, our study seeks to achieve several objectives. Firstly, we aim to determine the level of compliance with HH among HCWs in 4 Dutch general practice offices using alcohol-based hand rub dispensers equipped with a built-in electronic counter. Secondly, we aim to evaluate the application of HHE by quantifying the usage of hand alcohol. Lastly, we aim to identify both facilitators and barriers that influence the practice of HH among HCWs in their daily routines. By elucidating these factors, our study aims to contribute valuable insights into improving HH practices in general practice settings, ultimately enhancing patient safety.

## Methods

### Design, Patients, and Study Setting

An observational study was conducted in 4 general practice offices (GPO) in the northern Dutch province Groningen between October 2019 and February 2020 (before the COVID-19 pandemic). One of the 4 GPOs was able to repeat this study during the pandemic (November and December 2021). All hand-rub dispensers in these GPOs were replaced for 1 month with an electronic hand-rub dispenser with WiFi technology and encrypted communication (Ingo-man Weco dispensers; Ophardt Hygienetechnik, Issum, Germany). This system autonomously recorded dispenser activation information, including time, location, and the dispensed product’s volume. As such, it was exclusively a replacement of the dispensers, the workflow was unchanged. The mechanical hand-rub device dispenses 1.5 ml (adjustable) of hand sanitizer per full pull (exact volume of dispensed hand-rub is measured). Any number of pulls of the dispenser’s handle within no more than a 2-s interval between each pull is defined and recorded as 1 hand hygiene event (HHE). Dispensers were located in each room where patient care contact took place and were used by all HCWs according to their routine patient care visits or tasks. HCWs were classified as: general practitioners (GPs; including GPs in training), practice assistants (PAs), and nurse practitioners (NPs). Each room was exclusively used by 1 type of HCW. HCWs were asked not to wash hands (unless visibly soiled) but to use ABHR in their daily practice. HCWs were prior informed about the purpose of this study. The HH compliance rate was calculated according to the WHO strategy and criteria as hand disinfection in an opportunity for HH^8^ and for this study considered the ‘actual compliance’. The 5 HHE moments identified in this strategy include (1) prior to patient contact, (2) prior to a clean or aseptic procedure, (3) after contact with body fluid, (4) after patient contact, and (5) after contact with the patient environment. The fifth could not be assessed for the current study. Data were extracted from the following GP electronic patient systems: Promedico (ICT-services and consultancy, Utrecht, The Netherlands), and Omnihis (Omnihis B.V., ICT-services and consultancy, Bunnik, The Netherlands). We anonymously extracted the reasons for the GP contact from the systems, excluding any other patient-specific data. We had no access to the medical records of individual patients. In addition, all patient-directed procedures were recorded manually, and HH opportunities were assigned accordingly.

### Professional and Practice Characteristics

HCWs were asked to register the number of their daily patient care contacts, how many patients were seen with an indication for hand hygiene and how many HH moments were done (self-assessment compliance). After the observation period, to measure professional and practice characteristics, a questionnaire containing 7 items was used for each HCW (N = 36): age, gender, years of experience, form of GPOs practice, function of HCWs, training in IP and the preferred method of hand hygiene (soap and water, or alcohol-based hand rub (ABHR)) outside this study, that is, during their regular activities in daily life. The questionnaire on professional and practice characteristics also contained a small qualitative component, allowing participants to provide comments on facilitators and barriers to the performance of HH (participants could write their individual comments).

### Statistical Analysis

Data were analysed using R version 4.0.3. Descriptive statistics (frequency analysis and Chi squared tests) were applied where appropriate. A *P* value of <.05 was considered to be statistically significant.

## Results

### Study Population

Thirty-six HCWs were included across 4 different GPOs; 17 general practitioners, 15 practice assistants, and 4 nurse practitioners from 4 GPOs. The age of the HCWs ranged from 19 to 67 years (median = 47.0); 78% of the participants were female (Table 1). In total per year, the number of registered patients in the 4 GPOs varied from 2000 to 5000. The number of patients which has a consult varied from 7000 to 12 000/year.

**Table 1.** Baseline Characteristics of the Health Care Workers (N = 36).

	General practitioners	Nurse practitioners	Practice assistants
Characteristic	Median (IQR)	Median (IQR)	Median (IQR)
Age (years)	45 (28-67)	57 (53-65)	46 (19-63)
Work experience (years)	18 (1-40)	34.5 (16-47)	21 (1-40)
	N (%)	N (%)	N (%)
Gender			
Male	8 (47.1)	0	0
Female	9 (52.9)	4 (100)	15 (100)
GPs offices forms^a^			
HOED	9 (52.9)	2 (50.0)	10 (66.7)
Duo	3 (17.6)	1 (25.0)	2 (13.3)
Group	5 (29.4)	1 (25.0)	3 (20.0)
HCWs trained in IP			
Yes	3 (17.6)	0 (0.0)	1 (6,7)
No	14 (82.4)	4 (100)	14 (93.3)
Preferred method of hand hygiene			
Soap and water	9 (52.9)	1 (25.0)	7 (46.7)
ABHR	7 (41.2)	3 (75.0)	6 (40.0)
No preference	1 (5.9)	0 (0.0)	2 (13.3)

Abbreviations: ABHR, alcohol-based hand rub; GPs, general practitioners; HCWs, Health care workers; IP, Infection prevention.

a A HOED is an organization of legally distinctive GP practices which are working within a single building; A Duo practice is a single GP practice with 2 GPs; Group practices are organizations of collaborating GPs as 1 legal entity. This study consisted of 2 HOEDs.

### Hand Hygiene Compliance

**Figure 1.**
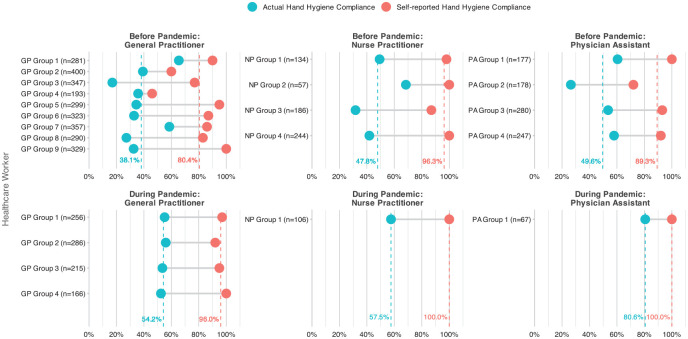
Hand hygiene compliance per healthcare worker. The dotted lines are the mean values per type of HCW (General Practitioner, Nurse Practitioner, or Physician Assistant). Behind each HCW is the number given of possible hand hygiene events. For this figure, the HWCs were split based the dispenser system used. HCWs were categorised into groups since the dispensers were room-based and not purely for individual use.

In the pre-COVID-19 study period, HH compliance was 41% (1786 out of 4322 opportunities. The mean HH compliance differed widely by profession: GPs had an actual compliance of 38% (1073/2819) where they self-reported 80%. Practice assistants had an actual compliance of 51% (447/882) where they self-reported 89%. Nurse practitioners had an actual compliance of 43% (266/621) where they self-reported 96%. GP office D, which performed the study before and during the pandemic, had an overall actual compliance of 42% (746/1790) prior to the pandemic, versus 56% (618/1096) during the pandemic, where they self-reported 91% and 97%, respectively (see Figure 1 and Table 2). Compliance did not differ significantly over the observation weeks for any of the GPOs.

**Table 2.** Hand Hygiene Compliance of Health Care Workers in General Practice Offices: General Practitioners, Nurse Practitioners, and Practice Assistants.

HCW type	HHE opportunities N	Compliance, actual %	Compliance, self-reported %	Compliance, difference %	Difference between HCW type (Chi^2^ test)
Before COVID-19					
GPs in GPO A	341/681	50	72	−22	
PAs in GPO A	107/177	60	100	−40	
NPs in GPO A	66/134	49	98	−49	
Total GPO A	514/992	52	87	−35	0.017
GPs in GPO B	128/540	24	66	−42	
PAs in GPO B	47/178	26	72	−46	
NPs in GPO B	39/57	68	100	−32	
Total GPO B	214/775	28	74	−46	0.531
GPs in GPO C	103/299	34	95	−61	
PAs in GPO C	150/280	54	93	−39	
NPs in GPO C	59/186	32	87	−55	
Total GPO C	312/765	41	92	−51	<0.001
GPs in GPO D	501/1299	39	89	−50	
PAs in GPO D	143/247	58	92	−34	
NPs in GPO D	102/244	42	100	−58	
Total GPO D	746/1790	42	91	−49	<0.001
TOTAL ALL GPO	1786/4322	41	86	−45	
During COVID-19					
GPs in GPO D	503/923	54	96	−42	
PAs in GPO D	54/67	81	100	−19	
NPs in GPO D	61/106	58	100	−42	
Total GPO D	618/1096	56	97	−41	<0.001

Abbreviation: GPO, general practice office; GPs, general practitioners; HCW, health care workers; HHE, hand hygiene event; NPs, nurse practitioners; PAs, practice assistants.

The differences between HCW type were tested using Chi^2^ tests.

According to the answers in the questionnaire (self-reporting), 17 HCWs have a slight preference to perform HH with soap and water (47.2%) and 16 HCWs (44.4%) – with ABHR (N = 33). Three HCWs have indicated that they have no preference in the method of HH (8.3%).

### Performance of Hand Hygiene Event

**Figure 2.**
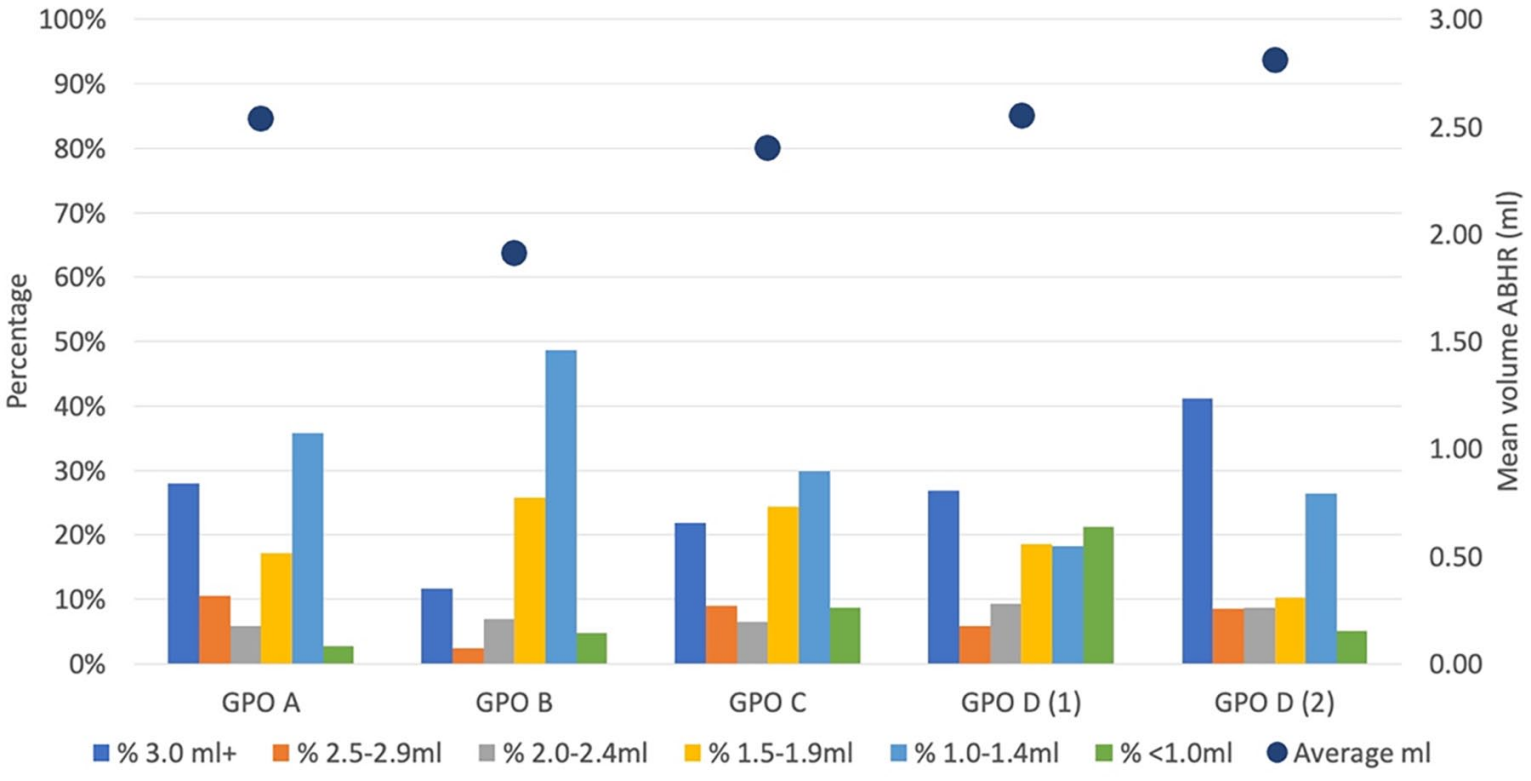
Hand hygiene performance per general practice offices. *GPO A, GPO B, GPO C, GPO D (1)* are general practice offices for COVID-19; *GPO D (2)*, during COVID-19; *ABHR*, alcohol-based hand rub.

All dispensers were set to give out 3 ml of ABHR after 2 consecutive full pulls. The mean volume of ABHR per HHE varied for GPO A to D: 2.54, 1.91, 2.40, and 2.55 ml, respectively. The mean volume of ABHR per HHE of GPs office D during the pandemic reached 2.81 ml, still remaining slightly below 3 ml. We observed that only 20% of HCWs from all GPOs before the pandemic took 3 ml of ABHR, while during the pandemic the used volume of the required 3 ml increased from 27% to 41% in GP office D. In the pre-pandemic period, 21% of the HCWs in GPO D used ABHR below 1 ml, and it decreased to even 5% in the post-pandemic period (Figure 2).

### Barriers and Facilitators of the HH Practices

Twenty-seven HCWs (75%) identified several facilitators and barriers influencing the use of ABHR compared to handwashing with soap and water (Table 3). ABHR was praised for its speed and convenience, as it requires no water and is easily portable, making it ideal for busy clinical settings. It was also perceived as more skin-friendly, preventing dryness associated with frequent handwashing. However, some HCWs noted that ABHR could feel less effective, particularly against certain viruses, and reported feeling less clean after use. Concerns about its higher cost and potential for unpleasant smells were also raised. Despite these barriers, ABHR’s strong endorsement in professional guidelines, especially for sterile procedures, and its proven efficacy in preventing cross-contamination were significant facilitators. Ultimately, personal preferences, such as product smell and feel, played a role in compliance, highlighting the need for diverse options to address individual needs and promote consistent hand hygiene practices.

**Table 3.** List of Facilitators and Barriers According to Experience of HCWs of GPOs for the Use of ABHR Versus Washing With Soap and Water.

Factor	Facilitators using ABHR	Barriers using ABHR
Application speed	Quick, no water needed	Drying effect, may require time to fully absorb
Hygiene	Cleaner, hygienic, does not dry out hands	Less effective against certain viruses, feels less clean
Smell	Some have a pleasant smell	Some have an unpleasant smell
Cost		More expensive than soap
Usability	Small and easily transportable, promotes habituation to use	Chemical, less standard than washing hands with soap
Professional practice	Recommended in guidelines (eg, before sterile procedures)	Not applicable for certain indications (eg, visibly soiled hands)
Efficacy	Prevents cross-contamination, faster use between patients	Ineffective against some bacteria, especially if GPOs not found
Preference	Individual preferences may favour scent and ease of use	Individual preferences may find residue unpleasant

Abbreviations: ABHR, alcohol-based handrub; GPOs, general practice offices.

## Discussion

Despite national and international guidelines with recommendations about infection prevention and control, HH compliance remains a challenge.^8,16-18^ To the best of the authors’ knowledge, this is the first study in the Netherlands to monitor HH performance electronically in GPs offices. Our study revealed notable areas of concern in the general practice setting, especially the low overall hand hygiene compliance compared to the self-reported compliance. While the mean volume of ABHR per HHE remained below 3 ml, an amount used for standardized testing and recommended by producers, the used amounts seem sufficient in most cases, with only some HCWs needing extra training to make them aware of the need for sufficient volume to completely cover their hands.

The recommended switch from hand wash to ABHR by the WHO was not followed by the majority of the health care workers in the participating GPOs. Examining the barriers and facilitators provides insight into potential interventions to enhance hand hygiene compliance.

Direct observation of HH helps to pinpoint areas of strength or weaknesses in HH behaviour, to identify the number of HH opportunities and their indications, to assess technique and to provide feedback to healthcare workers. Still, direct observation of HH in GPOs is challenging and is highly time-consuming. Specifically, the observer needs to be present with the general practitioner and patient in the same room and would need explicit permission, making covert direct observations impossible. In addition, the number of opportunities to be observed is fairly low at the GPOs.^17^ Electronic dispensers prove valuable in collecting continuous time trend data for usage frequency. However, it is important to note that dispensers only register use, and cannot determine whether hand hygiene is executed at the correct moment and/or was performed appropriately. Yet, electronic dispensers allowed real-time documentation of hand hygiene event without increasing the workload. Srigley et al^19^ reviewed impact on compliance and found an improvement in HH on entry to or exit from an area. Wang et al,^20^ in their recent review identify system-associated issues and challenges, including system accuracy, data integration, privacy and confidentiality, potential risks, usability, and associated costs and infrastructure improvements. Most studies look at electronic monitoring of HH in hospital settings. Implementation of this strategy in primary care has been little studied, which hinders comparison with the present study and urges for more focussed research in primary care.

The HCWs were aware of the fact that measuring hand hygiene was the goal of the study, so we hypothesized that the presence of the dispensers would create a permanent sense of being observed, leading to a so-called positive Hawthorne effect, despite HCWs probably getting used to being observed. This effect is therefore probably disappearing after some time. Consequently, we expected that in the first weeks of the study, compliance would be somewhat higher in GPOs and that it would normalize after a number of weeks. However, we saw the same use of ABHR over the weeks and compliance stayed at the same level.

HCWs were asked to primarily use ABHR, and soap and water only in specific situations. Despite this, some HCWs might have habitually used water and soap leading to an underestimation of HH compliance. Therefore, future research should include the measurements of using water and soap.

Electronically determined compliance averaged 41%, which is in accordance with 37% reported by Hilt et al^17^ in primary care and 40% reported by Erasmus et al^18^ in hospital care. HH compliance differed significantly between the various professionals, with GPs scoring lower than nurse practitioners (38% vs 43%). Practice assistants showed the highest compliance (51%). Allegranzi et al^21^ found baseline compliance of 55% for nurses and 44% for physicians in a global implementation of the WHO multimodal strategy in mixed-income countries. Erasmus et al^18^ similarly reported higher compliance among nurses (48%) than among physicians (32%). Bredin et al^22^ showed in their systemic review that the weighted pooled compliance rate for nurses was 52% (95% CI = 47-57) and for doctors was 45% (95% CI = 40-49). Group specific training programmes, institutional policies, and job duties may contribute to the observed differences.

HH compliance differed between GPOs, from 27% to 52%. This remarkable heterogeneity in HH compliance may reflect a local GPO HH culture and does not necessarily depend on function group. In literature, role models and behaviour of other HCWs significantly influences compliance rates of hand hygiene.^23,24^ The overall HH compliance within the GPO D was 42% pre-pandemic and rose to 56% during the pandemic. While only 1 out the 4 GPOs was able to repeat this study during COVID-19 pandemic, our results comply with other research showing that HH compliance increased during the pandemic.^25^

While HH compliance is of upmost importance, it lacks details about adequate performance of HH. The volume of handrub applied plays a critical role in bacterial reduction following hand rubbing, as it could be seen as a surrogate for covering the complete hand.^26,27^ Kampf et al^28^ demonstrated that a higher bacterial load reduction was reached by applying more handrub: using 1.1, 2, or 5 ml of the same ABHR resulted in 1.85, 3.35, and 3.58 log_10_ reduction, respectively. Each dispenser in our study dispensed 3 ml of ABHR after 2 consecutive full pulls. In the WHO Guideline on Hand Hygiene in Health Care^8^ the volume of ABHR is not specified. Both the EN 1500 European Norm (test method to evaluate the efficacy of a handrub) and the North American standard ASTM E−1174 require the application of 3 ml of handrub.^29,30^ Zingg et al^31^ showed that at least 2 ml of ABHR is needed to completely cover all hand surfaces, but 3 ml may be insufficient in the case of large hands. Presumably, a volume of 1 ml of ABHR cannot cover the entire hand surface. A recent Dutch study has shown that 2.25 ml ABHR is required for adequate coverage (82-90%) of both sides of the hand.^32^ The overall mean volume of ABHR in the present study was 2.44 ml, varying per HHE between 1.91 and 2.55 ml. The mean volume of ABHR measured before and during the pandemic rose from 2.552.81 ml. We can assume that the average volume ABHR used pre- and certainly during the pandemic was sufficient to cover most HCWs hands, if an adequate hand-rub technique was used. Some of the HCWs could benefit from additional training with regard to the importance of fully covering their hands during hand disinfection. These efficacies could be studied in future research.

In contrast to the low overall hand hygiene compliance of 41%, self-reported overall compliance was as high as 86%. This supports conclusions from other studies that observed practice is unrelated or weakly correlated to self-reported behaviour.^17,33,34^ This in turn can depend on different determinants, such as lack of knowledge, risk perception, and attitude.^34^ Moreover, Contzen et al^35^ showed that over-reporting of handwashing was associated with factors assumed to be involved in (1) socially desirable responding, (2) encoding and recall of information, and (3) dissonance processes. According to Diefenbacher et al,^36^ empathy of HCWs should be considered as an important factor in explaining differences between self-reported and observed HH compliance. It is expected that using self-reported behaviour could severely overestimate compliance, as has been illustrated in the present study as well.

Reasons which might explain suboptimal HH practices are multiple and may vary according to the setting and the resources available.^37^ For example, perception and knowledge of the transmission risk, HCWs self-efficacy beliefs, and the intention to perform HH.^38,39^ According to Erasmus et al, if people believe that their hand hygiene is much better than it is, they are likely to be oblivious to current campaigns that aim to increase hand hygiene behaviour by changing attitude.^31^ Also, access to supplies, simple instructions, and having or being ‘a good example’ are perceived most effective to improve HH compliance, as has been illustrated in a recent survey.^40^ They showed that medical staff tends to copy the HH behaviour of their superiors, leading to non-compliance when they observe non-compliance by others. We believe that in Dutch GPOs, PAs, and NPs might also be prone to follow their ‘leaders’ (GPs) in non-compliance with HH. General practitioners mentioned that their non-compliance was also associated with a lack of evidence that HH is effective in the prevention of HAIs.

We observed barriers of HH performance such as the perceived lack of time and product availability. Some GPs expressed doubts with regard to the use of ABHR due to ‘absence of pathogenic bacteria in their office’. Ahmadipour et al^41^ categorized barriers into 3 different causes for lack of compliance of hand hygiene: barriers related to individuals, barriers related to management, and barriers related to organizations. Future research should focus on these in primary care to gain more insight in these perceived barriers, perhaps with a focus on lack of knowledge of healthcare workers and healthcare workers’ improper attitude as we saw some of these individual barriers reflected in our research.

If the hands are not visibly soiled, national and international guidelines on HH^8,16^ prefer hand disinfection with ABHR over applying soap and water. However, half of HCWs in our study (self-reporting) preferred to use water and soap in their daily practice. Some HCWs were convinced that hand washing is the standard of care in the national guidelines, indicating that training is needed.

Drivers for better HH practice, mentioned were: HH with ABHR is quick, cleaner, easily applicable, and hygienic. ABHR is better to use for a sterile procedure, it prevents contamination and protects. It was interesting to see that smell and dry skin were mentioned as facilitators as well as barriers for HH practice and these statements remain individual.

Overall, our study shows that more interventions are needed to increase HH compliance and allow for the switch to ABHR.

### Limitations and Strengths

Compliance was calculated retrospectively based on data from dispensers and patient electronic dossier systems. We could not be sure that all HH moments are included, which might have led to an overestimation of HH compliance. The potential for recall bias is acknowledged; however, this risk is minimised as participants recorded data directly after each consultation block. Additionally, all HCWs were aware of the aim of the study, possibly leading to a positive Hawthorne effect, where individuals alter their behaviour due to the awareness of being observed. Our study population included only 36 professionals from 4 general practices in 1 geographical area, which might limit the generalizability.

Despite these limitations, we believe that our study is of significant value, as it is the first study to monitor HH performance electronically in Dutch general practice offices. The use of electronic dispenser allowed real-time documentation of a substantial number of hand hygiene events without increasing the workload of the HCWs. Information about barriers and facilitators in applying adequate HH practices, can help to guide future interventions.

## Conclusion

Hand hygiene (HH) compliance among healthcare workers (HCWs) in Dutch general practice offices (GPOs) was found to be suboptimal at 41%. Notably, general practitioners exhibited the lowest compliance rates, while practice assistants demonstrated the highest. Despite the mean volume of alcohol-based handrub (ABHR) per hand hygiene event (HHE) falling below European and the North American standards,^29,30^ it aligns with findings from field studies and was in an suitable range on most occasions.^31,32^ However, direct observation is necessary to confirm that an adequate hand-rub technique is being used to ensure complete hand coverage. Still, data on ABHR use (which could also be expanded to soap use) can be helpful to determine the success of interventions to increase compliance in the setting of GPOs.

To address deficiencies, multi-modal interventions are essential to enhance HH compliance and promote the adoption of ABHR over traditional handwashing with water and soap. The notably low compliance rates and inadequate ABHR volumes highlight the urgent need for targeted interventions and further research. Understanding the barriers to compliance and the specific requirements of GPs is critical for developing effective infection prevention and control (IPC) strategies in primary care. Future studies should focus on identifying practical and scalable solutions to improve HH practices, ensuring HCWs adhere to recommended guidelines to protect both patients and themselves.
